# Unexplained cyanosis revealing hepatopulmonary syndrome in a child with asymptomatic congenital hepatic fibrosis: a case report

**DOI:** 10.1186/1752-1947-7-120

**Published:** 2013-04-29

**Authors:** Atqah Abdul Wahab, Maryam Al-Mansoori, Mariam El-Hawli, Vishwanatha Kini

**Affiliations:** 1 Department of Pediatrics, Hamad Medical Corporation, P.O. Box 3050, Doha, Qatar; 2 Department of Radiology, Hamad Medical Corporation, P.O. Box 3050, Doha, Qatar

**Keywords:** Hepatopulmonary syndrome, Congenital hepatic fibrosis, Liver transplantation

## Abstract

**Introduction:**

Hepatopulmonary syndrome is a rare disease that affects patients of any age with acute or chronic liver disease. Liver transplantation is the only therapeutic option of proved benefit, and can result in substantial improvement or total improvement in postoperative gas exchange abnormalities.

**Case presentation:**

We report the case of a cyanotic 13-year-old Pakistani boy whose chest computed tomography scan showed normal lung fields and mediastinum with incidental findings of a prominent liver surface with a collateral vein connecting a portal cavernoma to the dilated terminal inferior vena cava. Sonography of his abdomen along with a portal venous Doppler study showed multiple collateral veins replacing the portal vein. A liver biopsy revealed congenital hepatic fibrosis. Contrast-enhanced echocardiography with agitated saline and a 99m Technetium-macroaggregated albumin perfusion lung scan confirmed intrapulmonary shunting. The patient underwent a successful liver transplantation that resulted in improved gas exchange.

**Conclusions:**

Hepatopulmonary syndrome should be included in the differential diagnosis of unexplained hypoxemia with an evaluation of possible portal hypertension or liver disease even in the absence of other clinical symptoms.

## Introduction

Hepatopulmonary syndrome (HPS) is a complication of portal hypertension defined by the presence of liver disease, hypoxemia, and evidence of intrapulmonary vascular dilatations (IPVD) producing intrapulmonary shunting [1]. The hallmark of HPS is the presence of IPVD, which may be secondary to portal hypertension producing a right-to-left intrapulmonary shunt [1,2]. The vascular dilatations cause over perfusion relative to ventilation, leading to ventilation-perfusion mismatch and hypoxemia. Liver transplantation is the only therapeutic option of proved benefit, and it can result in substantial improvement or total resolution in postoperative gas exchange [3]. However, the postoperative mortality rate of patients with severe hypoxemia before transplantation has been high [1]. Patients with a baseline PaO2 ≤50mm Hg have been associated with a poor survival rate [4]. We describe a rare case of a child presenting with unexplained hypoxemia in asymptomatic liver disease, revealing HPS and improved gas exchange post liver transplant.

## Case presentation

**Figure 1:**
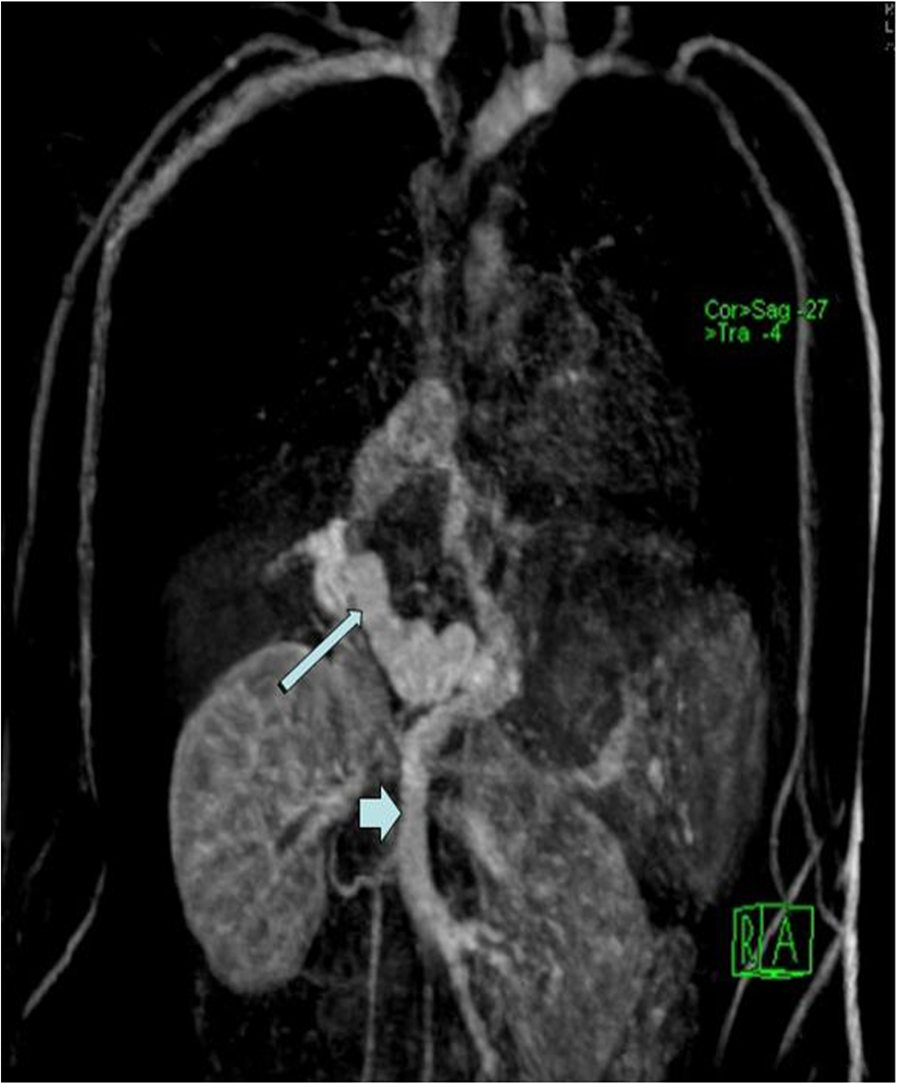
Coronal magnetic resonance imaging (MRI) scan of the chest and abdomen section: dilated portal vein (long arrow) and congested mesenteric veins (short arrow).

A 13-year-old Pakistani boy presented to our clinic with cyanosis, an intermittent cough, decreased exercise tolerance and episodes of bluish discoloration of his hands and feet that had been ongoing for one year. A clinical examination revealed clubbing and cyanosis with room air oxygen saturation of 88% in the supine position and 87% while sitting. There was no history of jaundice, cholangitis, hematemesis, melena or rashes. The chest was clear and there was no heart murmur. An abdominal examination revealed no abnormalities. There was no hepatosplenomegaly. Skin examination showed no stigmata of chronic liver disease. The patient had an increased alveolar-arterial gradient (A-a PO_2_ gradient) of 26mmHg and was started on two liters of oxygen that improved his oxygen saturation to 97%. His chest X-ray and echocardiogram were normal. Spirometry revealed normal percentages of predicted FEV_1_ (forced expiratory volume in one second) 90%, FVC (forced vital capacity) 86%, and FEV1/FVC 89%. Cardiac catheterization showed normal pulmonary pressure. His chest computed tomography (CT) scan was normal. An incidental finding of a prominent liver surface and collateral vessels connecting a dilated portal to the dilated terminal inferior vena cava (IVC) and mild splenomegaly was noted. Sonography of his abdomen along with the portal venous Doppler study showed a dilated portal vein. A magnetic resonance imaging (MRI) scan of his abdomen showed attenuated hepatic veins and normal intrahepatic portal vein branches with a maintained texture pattern. The main portal vein was replaced by multiple prominent collaterals transforming to a portal cavernoma (Figure 1).

**Figure 2:**
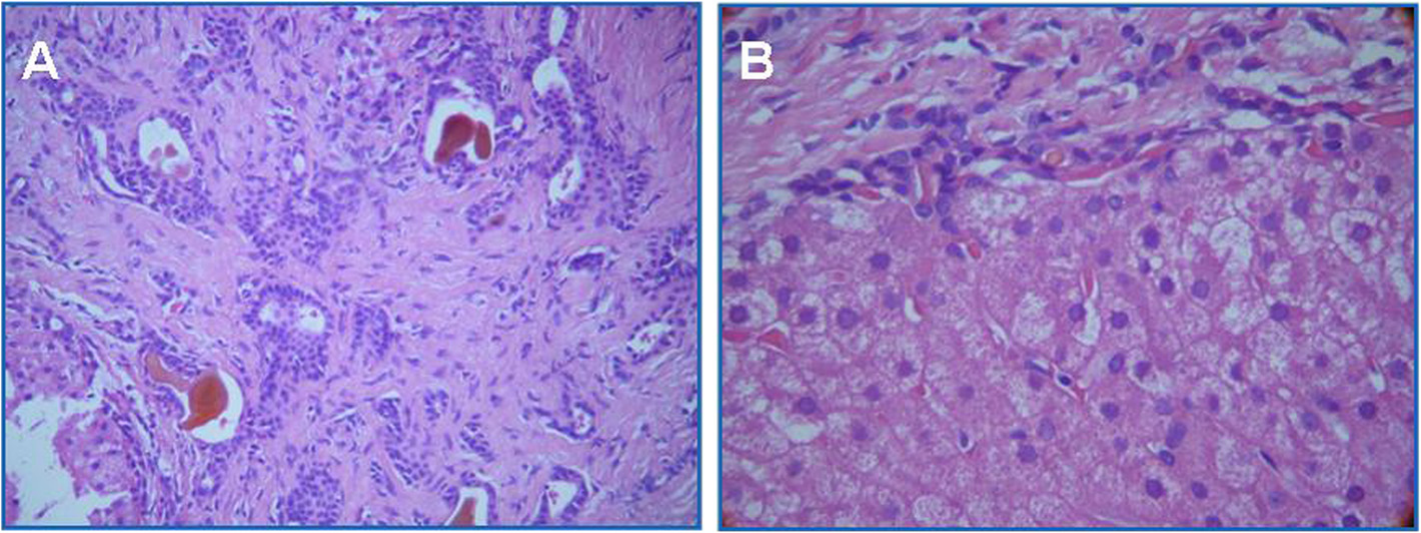
**Hematoxylin and eosin stain of liver tissue (400X magnification).** (**A**) Bile duct proliferation in the fibrosis areas with bile stasis and little inflammation. (**B**) Hepatocytes in lobules without significant inflammation or necrosis and surrounding portal fibrosis.

His liver function tests, coagulation profiles, serum ceruloplasmin, alpha-1 antitrypsin, antinuclear antibodies (ANA), and antineutrophil cytoplasmic antibodies (ANCA) were all normal. Endoscopy of his upper gastrointestinal tract showed no esophageal varices. An ultrasound-guided liver biopsy showed hepatocytes in lobules without significant inflammation or necrosis, surrounding portal fibrosis and bile duct proliferation in the fibrosis areas with bile stasis and little inflammation, all suggestive of congenital hepatic fibrosis (Figure 2A, B).

Contrast-enhanced echocardiography with agitated saline (CEE) and a 99m Technetium-macroaggregated albumin perfusion lung scan (^99m^Tc-MAA) were compatible with an intrapulmonary right-to-left shunt. CEE is considered diagnostic if the bubbles are in the left heart cavities at least three beats after their visualization in the right cavities, as was found in our patient [5]. ^99m^Tc-MAA is an injectable radiopharmaceutical used in nuclear medicine. It consists of a sterile aqueous suspension of Technetium-99m (^99m^Tc) labeled to human albumin aggregate particles in the pH range of 3.8 to 8.0. ^99m^Tc-MAA perfusion scanning is a more sensitive procedure as it allows quantification of the degree of intrapulmonary shunting. ^99m^Tc-MAA particles >20mm in diameter are entrapped in pulmonary vasculature and undergo decay [6]. In patients with a right-to-left shunt, the ^99m^Tc-MAA enters the systemic circulation and distributes to systemic organs, as was found in our patient.

The patient underwent a successful living-related liver transplantation with no serious complications. Histopathology of his liver resection confirmed congenital hepatic fibrosis. The patient required oxygen in the initial postoperative three months until his PO_2_ improved to 90mm Hg in room air and he did not require further oxygen therapy.

## Discussion

HPS is a rare complication of congenital hepatic fibrosis. Poor oxygenation with no major abnormalities detected from chest imaging and spirometry was the best diagnostic clue in this case.

HPS was first described in 1977 by Kennedy and Knudson [7]. The currently accepted diagnostic criteria for HPS are (1) presence of portal hypertension or liver failure, (2) decrease of arterial PO_2_ (PaO_2_ <70mm Hg, or increased age-corrected A-a PO_2_ gradient, and (3) presence of IPVD producing an intrapulmonary shunt. This is the hallmark of HPS and is probably secondary to portal hypertension and producing a right-to-left intrapulmonary shunt [1]. HPS primarily affects the peripheral (precapillary and capillary) branches of the pulmonary vascular tree at the lung bases. The most striking findings of the present case were digital cyanosis and clubbing with severe hypoxemia. Findings on physical examination of the chest were unremarkable. The patient did not elicit platypnea and orthodeoxia (reduced PO_2_ when changing from a supine to an upright position) that has been reported in the majority of patients with HPS [8].

A recent study reported the prevalence of HPS in 31 consecutive patients with noncirrhotic portal hypertension, in which only five patients had congenital hepatic fibrosis [9].

Congenital hepatic fibrosis is a developmental disorder that belongs to the family of hepatic ductal plate malformations and is characterized histologically by variable periportal fibrosis and irregularly shaped proliferating bile ducts. The clinical manifestation of congenital hepatic fibrosis is nonspecific, which makes the diagnosis of this disorder extremely difficult. The onset of symptoms is highly variable and ranges from early childhood to the fifth or sixth decade of life, although this disorder is diagnosed in most patients during adolescence or young adulthood, as was the case with our patient [10]. Although individuals with congenital hepatic fibrosis rarely develop cirrhosis, they may still face complications of portal hypertension, particularly bleeding varices. In contrast to our patient, a recent case was reported of HPS in a patient with symptomatic congenital hepatic fibrosis with severe portal hypertension and large esophageal varices who underwent a successful liver transplant [11].

At present, there is no effective medical therapy considered useful in the management of HPS. Liver transplantation has emerged as a therapeutic option of proved benefit, and it can result in significant improvement or complete resolution in hypoxemia [3]. This resolution may take a relatively short time, three months as in our patient, or over a year as reported in some cases [12]. A recent study reported that living-related donor liver transplantation in children was associated with a high survival rate, and it included three cases of patients with congenital hepatic fibrosis [13].

## Conclusions

HPS should be considered in the differential diagnosis in patients with unexplained hypoxemia, and possible portal hypertension or liver disease should be evaluated even in the absence of clinical symptoms.

## Consent

Written informed consent was obtained from the patient’s next-of-kin for publication of this case report and any accompanying images. A copy of the written consent is available for review by the Editor-in-Chief of this journal.

## Competing interests

The authors declare that they have no competing interests.

## Authors’ contributions

AA and MA followed the patient, collected the data, reviewed the literature, collected all information, and wrote the manuscript. ME contributed to patient management and data collection. VK participated by providing radiographic reports, reviewing the literature and contributing to theoretical interpretation. All authors read and approved the final version of the manuscript.
